# Identifying plant traits to increase wheat yield under irrigated conditions

**DOI:** 10.1016/j.heliyon.2024.e31734

**Published:** 2024-05-22

**Authors:** Arezoo Abidi, Afshin Soltani, Ebrahim Zeinali

**Affiliations:** Department of Agronomy, Gorgan University of Agricultural Sciences and Natural Resources, Gorgan, 4918943464, Iran

**Keywords:** Wheat improvement, Traits, Yield, Crop modelling, Nitrogen, Water

## Abstract

Crop models have frequently been used to identify desired plant traits for rainfed wheat (*Triticum aestivum* L.). However, efforts to apply these models to irrigated wheat grown under non-limiting water and nitrogen conditions have been rare. Using simulation models to identify plant traits that impact yield can facilitate more targeted cultivar improvement and reduce time and cost. In this study, the SSM-iCrop model was employed to identify effective plant traits for increasing the yield of irrigated wheat in four distinct environments in Iran. A comprehensive range of traits related to phenology, leaf area development, dry matter production, and yield formation, which exhibited reported genetic variation, were tested. The impact of these traits on yield showed slight variation across different environmental zones due to genetic × environment interaction. However, across all environments, modifying current cultivars to increase radiation use efficiency (RUE) resulted in a 19 % increase in yield, accelerating leaf area development led to a 10 %–15 % increase, lengthening the grain filling period resulted in a 14 % increase, and extending the vegetative period led to a 6 % increase. These improvements were all statistically significant. Considering that longer duration cultivars may disrupt cropping systems and the need to develop simple methods for targeting and phenotyping RUE, faster leaf area development was found as the most promising option to increase irrigated wheat yield under optimal water and nitrogen management within a short time frame. It should be noted that cultivars with modified traits needed higher water and nitrogen inputs to support increased yields. These findings can be applied to select desirable key traits for targeted breeding and expedite the production of high-yielding cultivars of irrigated wheat in various environmental zones. The potential for further improvement through combined traits requires further investigation.

## 1 Introduction

To meet the growing demand for human food and livestock feed, it is crucial to enhance global crop production by 60–110 % between 2005 and 2050 [1]. Expanding agriculture to increase crop production is not a viable option due to limited land and water resources, as well as the significant environmental costs associated with such expansion [2]. Therefore, the focus is on increasing crop yield per unit area. This can be achieved in two ways: (a) by increasing the crop yield potential [3] and (b) by reducing the yield gap between farmers' actual yield and the yield potential [4]. The yield potential refers to the maximum yield achievable by current adapted cultivars under optimal crop management conditions, where limitations caused by water, nutrients, pests, diseases, and weeds are negligible [4].

In recent years, there has been a significant focus on improving the physiological traits of crop plants to enhance their yield potential [5]. The initial step in breeding programs aimed at improving physiological traits is to identify effective traits that can increase yield potential [6,7]. The identification of such traits simplifies the process of developing new cultivars with higher yields [8]. While the evaluation and discovery of new traits for increased yield is a national or international concern, it requires local solutions due to the interaction between genotype and environment. The differences observed between two genotypes in a field experiment depend on the applied management practices and the specific environment (location and/or year) of the field trial. Genotypic differences may be negligible under one set of management practices or environment, but significant under another. Consequently, numerous field experiments are necessary to assess a specific trait. However, conducting an adequate number of field experiments to address geospatial responses is impractical due to the extensive observations required to capture temporal and geographical variability [9].

One cost-effective and rapid method for identifying and evaluating the effects of modifying physiological traits on yield is the use of plant simulation models. Crop models serve as valuable tools for assessing genotype × environment × management interactions, provided they capture the complex relationships between genetics, management practices, soil conditions, and weather factors that influence crop yield and quality [9]. This approach enables a large number of virtual experiments to be conducted across different years and locations using fewer resources [7,8]. In the past, many researchers have utilized plant simulation models to investigate the impact of plant physiological traits on yield in various crops (e.g. Refs. [10,11]).

Most studies conducted to identify desirable traits for improving wheat (*Triticum aestivum* L.) yield have focused on rainfed conditions, particularly traits related to drought tolerance (e.g. Refs. [[12], [13], [14]]). For instance Ref. [12], used a wheat crop model to identify plant traits that could increase winter wheat yield in semiarid and subhumid environments in the US. Similarly, Senapati et al. [13] assessed drought-tolerant traits during reproductive development to enhance wheat yield under climate change in Europe. However, studies aimed at identifying and evaluating desirable traits for increasing wheat yield under irrigated conditions are scarce, with only three studies found [[15], [16], [17]]. Moreover, these studies were limited in terms of the number of traits (i.e., 2–4) they evaluated.

Considering that the mechanisms of yield formation differ under irrigated conditions with optimal water availability, it is intriguing to conduct similar evaluations under irrigated conditions to determine the most promising physiological aspect. Furthermore, in the majority of previous evaluations of desired plant traits, optimal nitrogen conditions were assumed, and soil and plant nitrogen sub-models were disabled (e.g. Refs. [15,17]). However, wheat yield under non-limiting water conditions is heavily dependent on nitrogen accumulation, and achieving high yields is possible only if the crop accumulates sufficient nitrogen [18]. Therefore, it is critical to include physiological traits associated with nitrogen accumulation in the assessment of desired plant traits under well-irrigated conditions.

Therefore, this research aims to identify and evaluate the effects of modifying plant traits on improving the yield of irrigated wheat in different climatic zones. The evaluations will incorporate soil nitrogen simulation, with an optimal fertilization routine implemented. For this purpose, the SSM-iCrop simulation model [7,19,20] will be utilized to conduct simulation experiments. This model has been extensively tested and applied in numerous studies, including the assessment of desired plant traits in various grain crops such as wheat (e.g. Refs. [14,21]), maize (e.g. Ref. [22]), and soybean (e.g. Ref. [23]).

## 2 Materials and methods

### 2.1 Locations

Four weather stations were selected in key wheat production areas of Iran, each representing a different climate zone. The selection of these stations was based on the findings of a previous study Zahed et al. [24], which involved the preparation of a distribution map of irrigated wheat at the national level in Iran. This study identified the most significant climatic zones for wheat cultivation and determined the representative weather stations for these zones. Using the GYGA-ED climatic zoning method [25], it was revealed that 40 % of the area under irrigated wheat cultivation in Iran is located in four climatic zones: 8003, 4003, 5003, and 6102. These climatic zones account for 14.3 % (8003), 12 % (4003), 9.3 % (5003), and 4.3 % (6102) of the total area under irrigated wheat cultivation in the country.

**Fig. 1:**
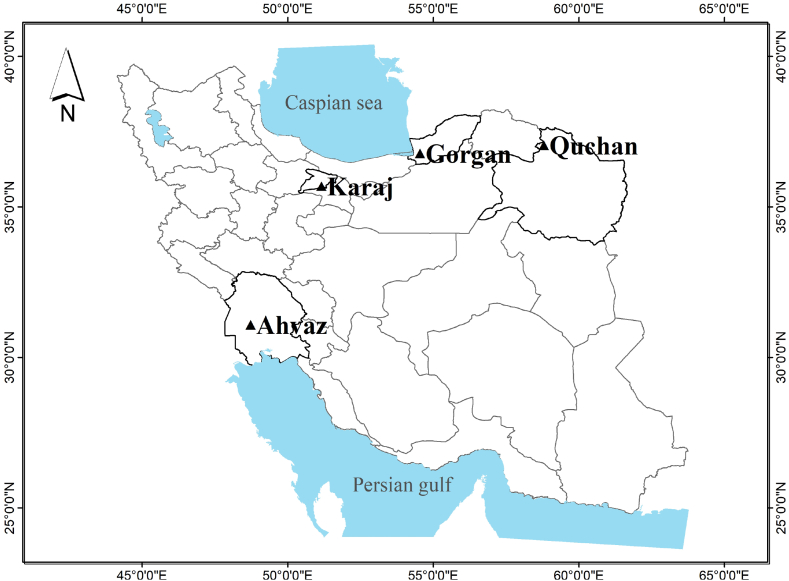
The weather stations (Ahvaz, Gorgan, Quchan and Karaj) that were used in the current study.

The selected weather stations are as follows: Ahvaz, representing climatic zone 8003, located in the south-western part of Iran (48.55° N, 31.25° E); Gorgan (Hashemabad), representing climatic zone 6102, situated in the north-eastern region of Iran near the Caspian Sea (54.26° N, 36.85° E); Karaj, representing climatic zone 5003, located in the north-central part of Iran (50.95° N, 35.81° E); and Quchan, representing climatic zone 4003, situated in the north-eastern region of Iran (58.50° N, 37.07° E) (Fig. 1).

**Fig. 2:**
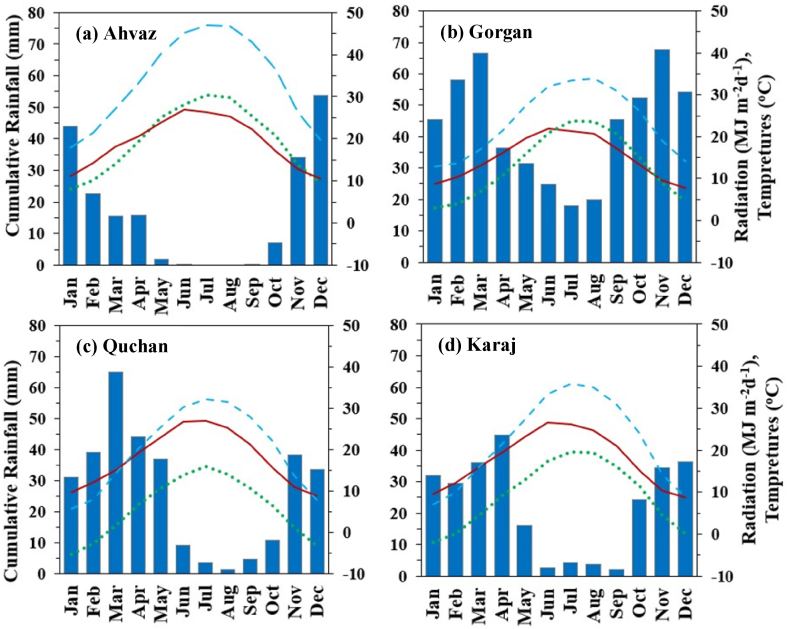
Monthly solar radiation (–––––), maximum (- - - -) and minimum (… …) temperature and rainfall (bars) for weather stations: Ahvaz (a), Gorgan (b), Quchan (c) and Karaj (d) based on weather records from 2000 to 2015.

The climatic conditions of the four locations are indicated in Fig. 2abcd. These climate indices represent 15-year averages (2000–2015) for their respective areas. The sowing time for wheat varied across the locations, ranging from mid-October to late December. In Ahvaz and Gorgan, which experience mild winters, spring wheat cultivars are sown from late November to late December. On the other hand, in Quchan and Karaj, which have cold winters, winter-type cultivars are sown from mid-October to mid-November. The average daily temperature during the growing season was 23 °C in Ahvaz, 15 °C in Gorgan, 9 °C in Quchan, and 12 °C in Karaj. The cumulative rainfall throughout the growing season (November to June) amounted to 151 mm in Ahvaz, 311 mm in Gorgan, 305 mm in Quchan, and 236 mm in Karaj. Moreover, the total intercepted solar radiation during the growth season (from sowing to physiological maturity) was 627 MJ m^−2^ in Ahvaz, 792 MJ m^−2^ in Gorgan, 1074 MJ m^−2^ in Quchan, and 956 MJ m^−2^ in Karaj.

### 2.2 Crop model

For this study, the SSM-iCrop model was used for crop simulation [7,19]. The initial SSM model for wheat was developed by Ref. [26] to investigate the impact of temperature and solar radiation on the growth and yield of the crop. Subsequently, this model was expanded to incorporate water limitation [27] and nitrogen limitation [28] effects on wheat growth and yield. [19]. further adapted the model to include more intricate features of the crop system.

The SSM-iCrop model employs the concept of biological days to simulate phenology. Biological days are calculated for each day based on temperature, day length, and vernalization, and are adjusted for water limitation. A biological day represents optimal conditions for plant development, where the growth stages occur in the minimum duration with maximum development rate [7].

To simulate leaf area expansion, the model utilizes the phyllochron concept, which calculates the daily increase in the number of leaves on the main stem [19], and the plant leaf area vs main stem leaf number allometry. The model also considers the effects of water and nitrogen availability on plant leaf area development [7,29], as well as the impact of extreme temperatures (both freezing and high temperatures) on leaf area development.

Dry matter production is calculated based on the intercepted photosynthetically active radiation (PAR) and the radiation use efficiency (RUE), which is adjusted for drought and temperature effects. Yield formation is based on a modified concept of linear increase in harvest index, as detailed by Soltani et al. [29]. The seed growth rate is determined by this concept but is limited by the rate of daily mobilized dry matter from vegetative organs and the current photosynthesis rate of the crop. The slope of the linear increase in harvest index is modified based on the pre-seed growth conditions experienced by the crop [19].

The SSM-iCrop model requires soil and weather information, including daily minimum and maximum temperature, solar radiation, and precipitation, to simulate plant growth, development, and yield. If necessary, the model incorporates water balance in the soil and nitrogen balance in both the soil and plant. It simulates soil water balance and its components, such as soil evaporation, plant transpiration, runoff, deep drainage, and the impact of water limitation on plant processes. Similarly, the model simulates the nitrogen balance in the soil by calculating volatilization, denitrification, leaching, N uptake, and mineralization. For more technical details about the model, please refer to Refs. [7,19].

The SSM-iCrop model for wheat has been parameterized and tested in Iran under a wide range of environmental and crop growth conditions [19,30,31], so a separate evaluation and testing of the model are not presented here. The model testing results have demonstrated satisfactory performance. According to the latest evaluation [31], the Normalized Root Mean Square of Error (nRMSE) was 6 % for days to maturity, 19 % for LAI at anthesis, 12 % for crop total dry mass, 12 % for grain yield, and 16 % for total accumulated nitrogen. The correlation coefficient (r) between simulated and observed data was 0.96 for days to maturity and 0.8 for LAI at anthesis, crop total dry mass, total accumulated nitrogen, and 0.85 for grain yield. Furthermore, the model has been successfully tested and utilized for wheat in previous studies conducted in other locations, such as the US [12,32], Middle East and North Africa [14,21,33,34], and Europe [35,36]. The model can be accessed at: https://sites.google.com/view/ssm-crop-models.

### 2.3 Simulations

The SSM-iCrop model requires weather data as input for simulation. In this study, climate data, including daily rainfall, minimum and maximum temperatures, and sunshine hours, were obtained from the Iran Meteorological Organization for a 15-year period (2000–2015). Daily solar radiation was calculated using the Angstrom equation based on the sunshine hours. Soil information was also needed for each weather station buffer, which was retrieved from the HC27 Soil Database [37]. The quality of the information in this database has been previously verified [38]. Soil for each location was as follows: Ahvaz had a loam soil with low fertility (23 kg N ha^−1^ at the beginning of simulation) and a soil depth of 120 cm, while Gorgan, Quchan, and Karaj had clay soils with medium fertility (30 kg N ha^−1^ at the beginning of simulation) and a soil depth of 120 cm. Supplementary Information (Table S1) provides more details on soil profile characteristics.

To ensure that the water deficit does not limit the growth and yield of wheat crops under irrigated conditions, the model was set to automatically irrigate the crops whenever 40 % of the available water in the root zone was depleted. Similarly, to prevent nitrogen limitation from affecting crop growth and yield, an automatic nitrogen fertilization method was employed. This method applied nitrogen fertilizer at sowing to bring the soil nitrogen to 23 kg ha^−1^ if the available nitrogen in the top 60 cm of soil was less than 23 kg ha^−1^. From emergence to anthesis, if the nitrogen in the soil solution in the top 60 cm decreased to less than 10 kg ha^−1^, a top dressing of either 50 or 60 kg ha^−1^ nitrogen (depending on the climatic zone) was applied, provided that there was at least 47 mm of available water in the plant root zone.

The date of wheat sowing in the weather station buffer zones was another necessary input for running the model. Wheat crops were virtually sown on the 5th day of a 5-day period without precipitation in the fall when the average temperature of the 5-day period dropped below 15 or 18 °C, depending on the region [24]. A plant density of 330 plants per square meter was used in all selected areas.

The results presented in this study primarily focus on grain yield. For each simulation, the average yield, coefficient of variation, and relative change in yield were calculated. The significance of the difference between the modified cultivars and the standard cultivars was tested using a *t*-test.

### 2.4 Designing modified cultivars

The differences between cultivars in the model are reflected in the values of the parameters used to define various processes. SSM-iCrop model of the present study utilized 61 crop parameters (Table S2 in SI). These parameters are not explicitly separated into species- and cultivar-related categories. However, the number of parameters required to differentiate between common cultivars is much smaller than the total number of parameters. For instance, only nine parameters were sufficient to distinguish between wheat cultivars, primarily phenology parameters in the study of [30]. The limited number of cultivar-dependent parameters does not imply the absence of genetic variation for other parameters. Rather, it indicates that conventional cultivars tend to share similar estimates for many parameters. If genetic variation exists, all parameters can be adjusted for new cultivars. It must be noted that there are certain differences between cultivars that are not considered by the model. For instance, variations in disease resistance among cultivars are not accounted for, as the model assumes effective disease control measures.

Table 1 presents the hypothetical improved cultivars created for this study by modifying the model parameters. The rationale behind creating these specific virtual cultivars is as follows. Firstly, all 61 model parameters were modified in both positive and negative directions, and the changes that positively impacted crop yield were identified. Secondly, literature was reviewed to identify the parameters from the previous step that exhibited significant genetic variation. Lastly, the parameters identified in the second step were modified proportionally based on the genetic variation found for each parameter. In each virtual cultivar, one plant trait (parameter) was modified, while the other plant parameters were kept constant and identical to the current local cultivars (standard cultivars).

**Table 1:** The crop parameters (traits) that had been modified in SSM-iCrop model, and the assumptions behind the traits to increase yield by their modification. The value of traits in standard cultivars (STD), range of variability of the parameters, and the percentage changes applied (%) to the STD cultivar. Acronym is the symbol used for the crop parameter in the model and the modified virtual cultivar in the present study.

Acronym	Description and assumption behind the modification of the trait	STD	Range	Ref	% of change
PHYL	Lower phyllochron. Phyllochron defines the interval between the appearances of successive leaves on the plant stem. Decreasing phyllochron results in faster leaf emergence and leaf area development which in turn increases radiation interception and dry matter production.	95 (^o^C per leaf)	70–120	[39]	−20 %
PLAPOW	Faster leaf area expansion by increasing the coefficient (exponent; y = x^b^) in the power relationship between plant leaf area and main stem node number. An increase in the PLAPOW will increase the potential leaf area development rate, but the actual rate also depends on water and nitrogen limitation.	2.43	1.7–2.92	[15]	+20 %
RUE	Increasing radiation use efficiency under optimal conditions. An increase in RUE results in a higher crop dry matter production.	2.42 (g MJ^−1^)	1.11–2.93	[40]	+20 %
FRTRL	Higher fraction of total crop mass at the beginning of seed growth which is available for transfer to the grains.	0.22	0.1–0.35	[41]	+20 %
PDHI	Higher the slope of linear increase in harvest index during seed filling period. Higher PDHI results in higher grain filling rate.	0.019 (g g^−1^)	0.011–0.020	[42]	+20 %
PPSEN	Higher sensitivity to photoperiod. PPSEN is the slope of the relative response of development to photoperiod. Increasing this trait will increase the duration of the vegetative growth period.	0.001467	0.00272–0.00691	[43]	+100 %
bdANTPM	Longer duration from anthesis to physiological maturity. Increasing this trait will increase grain ﬁlling duration.	34 (day)	30–43	[44]	+20 %
SLNG	Higher specific leaf N in green leaves results in more nitrogen accumulation in leaves for later possible translocation to the grains.	1.8 (g m^−2^)	4.92–7.64	[45]	+25 %
SLNS	Lower specific leaf nitrogen in senesced leaves increases the amount of N available for translocation to the grains from the leaves.	0.4 (g m^−2^)	0.18–1.89	[46]	−25 %
SNCG	Higher stem nitrogen concentration in green stems. This will increase the accumulation of nitrogen in the stems that can be translocated to the grains if necessary.	0.019 (g g^−1^)	0.006–0.031	[46]	+25 %
SNCS	Lower stem nitrogen concentration in senesced stems which increases the amount of nitrogen available for translocation to the grains from the stems.	0.0095 (g g^−1^)	0.002–0.027	[46]	−25 %
MXNUP	Higher maximum rate nitrogen uptake. In conditions where demand for nitrogen by the crop is higher than MXNUP, the actual rate of nitrogen accumulation is limited to this maximum limit.	0.6 (g N m^−2^ d^−1^)	0.6–0.9	[16]	+25 %

## 3 Results

### 3.1 Grain yield of standard (current) cultivars

The average yield of the standard cultivars (STD) was 680 g m^−2^ for Ahvaz, 738 g m^−2^ for Gorgan, 914 g m^−2^ for Quchan, and 863 g m^−2^ for Karaj. The coefficient of variation for both improved and standard cultivars ranged from 10 to 13 % over the 15-year simulation period (Table 2). It is important to note that all simulated yields reported in this study are based on dry weight. Additionally, it should be mentioned that the yields of STD cultivars were 10–16 % higher when assuming non-limited nitrogen by deactivating the plant and soil nitrogen sub-models in the model, i.e., the yields were 768 g m^−2^ for Ahvaz, 815 g m^−2^ for Gorgan, 1061 g m^−2^ for Quchan, and 965 g m^−2^ for Karaj. However, achieving these potential yields would require applying large amounts of nitrogen, which is neither economically nor environmentally justifiable. The implemented automatic nitrogen application method resulted in 3–5 fertilizations, which is a common practice in wheat cultivation.

**Table 2:** Simulated crop Grain yield (g m^−2^) in modified cultivars in comparison with standard cultivar in Ahvaz, Gorgan, Quchan and Karaj.

Cultivar	Ahvaz				Gorgan			
	Yield (g m^−2^)		Yield change		Yield (g m^−2^)		Yield change	
	Mean	CV	Mean g m^−2^	Mean %	Mean	CV	Mean g m^−2^	Mean %
STD	679.9	13.0	–	–	738.0	12.3	–	–
PHYL	736.5	13.0	56.6	8.3***	814.9	12.1	76.9	10.4***
PLAPOW	753.2	12.2	73.2	10.8***	855.7	12.2	117.7	16.0***
RUE	812.4	12.6	132.5	19.5***	887.0	11.7	149.0	20.2***
FRTRL	705.5	13.2	25.6	3.8***	771.0	11.9	33.0	4.5***
PDHI	700.6	11.8	20.7	3.0***	763.1	10.8	25.1	3.4***
PPSEN	715.7	11.6	35.7	5.3**	774.5	12.9	36.5	4.9**
bdANTPM	781.4	11.4	101.4	14.9***	842.8	10.4	104.8	14.2***
SLNG	678.1	12.8	−1.8	−0.3^NS^	744.2	12.4	6.3	0.8 ^NS^
SLNS	681.9	13.1	1.9	0.3***	739.8	12.3	1.8	0.2***
SNCG	689.4	12.9	9.5	1.4**	748.8	12.5	10.8	1.5***
SNCS	685.2	13.1	5.2	0.8^NS^	740.9	12.3	2.9	0.4**
MXNUP	680.0	13.0	0.1	0.0^NS^	738.6	12.3	0.6	0.1 ^NS^
Cultivar	Quchan				Karaj			
	Yield (g m^−2^)		Yield change		Yield (g m^−2^)		Yield change	
	Mean	CV	Mean g m^−2^	Mean %	Mean	CV	Mean g m^−2^	Mean %
STD	913.8	11.4	–	–	863.1	10.7	–	–
PHYL	1020.9	10.5	107.1	11.7***	943.5	10.9	80.4	9.3***
PLAPOW	1080.4	10.6	166.6	18.2***	977.7	11.7	114.6	13.3***
RUE	1075.0	11.6	161.1	17.6***	1019.3	9.7	156.2	18.1***
FRTRL	955.9	11.0	42.1	4.6***	899.2	10.4	36.1	4.2***
PDHI	922.5	10.9	8.7	1.0 ^NS^	890.6	9.8	27.5	3.2***
PPSEN	999.1	9.9	85.3	9.3***	884.1	10.3	21.0	2.4**
bdANTPM	1026.8	11.1	113.0	12.4***	983.7	9.6	120.6	14***
SLNG	923.0	12.3	9.2	1.0 ^NS^	860.3	11.4	−2.8	−0.3 ^NS^
SLNS	916.6	11.5	2.8	0.3**	864.5	10.7	1.4	0.2***
SNCG	931.3	11.6	17.5	1.9**	867.8	10.8	4.7	0.5*
SNCS	918.7	11.3	4.9	0.5**	865.9	10.7	2.8	0.3**
MXNUP	914.9	11.4	1.1	0.1 ^NS^	864.6	10.7	1.5	0.2 ^NS^

NS: not significant; *, **, and ***: Significant at α = 0.05, 0.01, and 0.001.

The variations in crop yield among the standard cultivars (ranging from 680 to 914 g m^−2^) in different climatic zones studied can be attributed to differences in cumulative intercepted solar radiation during the growing season. In the warmer climatic zones of Ahvaz and Gorgan, the plant growing seasons were shorter, resulting in less cumulative intercepted solar radiation by the plant canopy throughout the growing season (from sowing to harvest) compared to the colder climate zones of Quchan and Karaj. The total cumulative intercepted radiation during the wheat growing season was 627 MJ m^−2^ in Ahvaz, 792 MJ m^−2^ in Gorgan, 956 MJ m^−2^ in Karaj, and 1074 MJ m^−2^ in Quchan (Tables S3 and S4 in Supplementary Information). Considering the high correlation between the total cumulative intercepted radiation and grain yield, the crop yield of standard cultivars in zones with warmer climates and shorter growing seasons (Ahvaz and Gorgan) was lower than in zones with cooler climates and longer growing seasons (Quchan and Karaj) (Table 2).

### 3.2 Modified cultivars

**Fig. 3:**
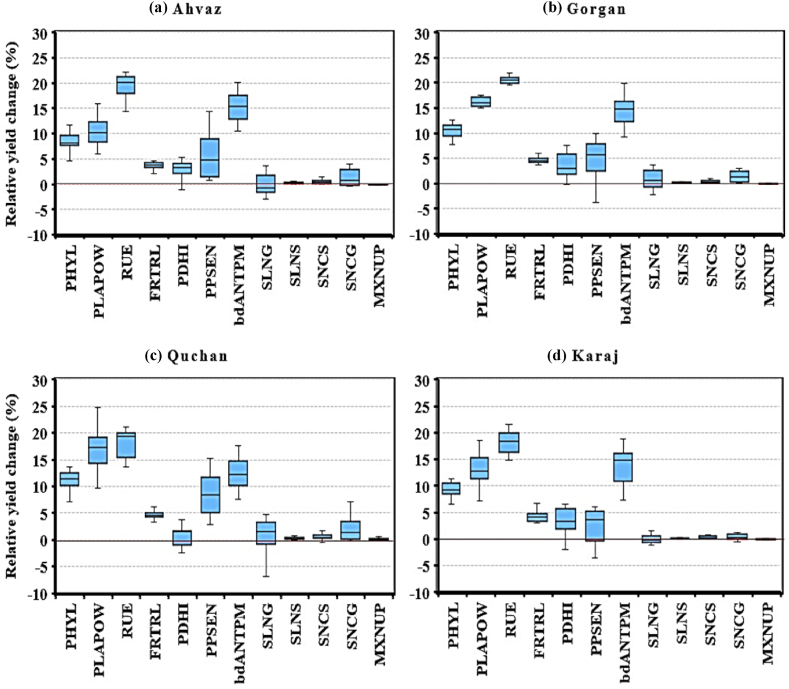
Simulated relative change (%) in grain yield by changing physiological traits (see Table 1 for definition) for each weather station: Ahvaz (a), Gorgan (b), Quchan (c) and Karaj (d). Simulated long-term average control grain yields were 680 g m^−2^ for Ahvaz, 738 g m^−2^ for Gorgan, 914 g m^−2^ for Quchan and 863 g m^−2^ for Karaj. Box boundaries indicate the 25th and 75th percentiles, the line within the box marks the median and the boundaries of the lower and upper whiskers are the minimum and maximum values of the data set, respectively.

The yield increase observed as a result of a 20 % reduction in phyllochron (PHYL) and a 20 % increase in PLAPOW was significant and notable in all zones. The reduction in PHYL led to an 8 % increase in yield in Ahvaz, 10 % in Gorgan, 12 % in Quchan, and 9 % in Karaj (Table 2 and Fig. 3abcd). On the other hand, increasing PLAPOW resulted in an 11 % yield increase in Ahvaz, 16 % in Gorgan, 18 % in Quchan, and 13 % in Karaj, compared to the standard cultivar. Across all zones, the effect of selecting PLAPOW had a greater impact than that of PHYL.

A 20 % increase in RUE compared to the control resulted in a yield increase of 20 % in both Ahvaz and Gorgan, and 18 % in both Quchan and Karaj (Table 2 and Fig. 3abcd). It was observed that the highest yield increase due to changes in plant traits has belonged to RUE. However, in Quchan, the yield increase resulting from modifications in PLAPOW was also 18 %, which was similar to the RUE-modified cultivar, and there was no significant difference in grain yield between the RUE and PLAPOW cultivars in this location.

The FRTRL cultivar exhibited a 4 % higher yield in both Ahvaz and Karaj, and 5 % in both Gorgan and Quchan, compared to the control cultivar, and this difference was statistically significant (Table 2 and Fig. 3abcd). Additionally, a 20 % increase in the slope of the linear increase in harvest index (PDHI) resulted in a 3 % yield increase in Ahvaz, Gorgan, and Karaj, compared to the control cultivars. The effect of modifying this trait on grain yield was significant in selected locations, except for Quchan (Table 2 and Fig. 3c).

In the present study, a 100 % increase in PPSEN (photoperiod sensitivity coefficient) resulted in an extended vegetative period of 5–7 days and a grain yield increase of 2–9 % in the studied zones. The findings showed a significant increase in grain yield in different zones when the vegetative period was extended through the PPSEN cultivar. The grain yield increased from 680 (in STD cultivar) to 716 g m^−2^ (5 %) in Ahvaz, from 738 to 775 g m^−2^ (5 %) in Gorgan, from 914 to 999 g m^−2^ (9 %) in Quchan, and from 863 to 884 g m^−2^ (2 %) in Karaj (Table 2 and Fig. 3abcd).

Increasing the duration of the grain filling period (bdANTPM cultivar) by 20 % resulted in a lengthening of the grain filling period by approximately 7–8 days (calendar days) compared to the standard cultivars. This change led to an increase in the growing season period from 129 days to 136 days in Ahvaz, from 187 days to 195 days in Gorgan, from 251 days to 258 days in Quchan, and from 222 days to 229 days in Karaj. The extension of the grain filling period was associated with an increase in grain yield, estimated to be 15 % in Ahvaz, 14 % in Gorgan, 12 % in Quchan, and 14 % in Karaj (Table 2 and Fig. 3abcd).

Altering nitrogen-related traits by 25 % had a minor effect on grain yield. The changes in wheat grain yield in response to a 25 % increase in specific leaf N in green leaves (SLNG) and nitrogen concentration in green stems (SNCG), a 25 % decrease in specific leaf N in senescent (yellow) leaves (SLNS) and nitrogen concentration in senescent stems (SNCS), and a 25 % increase in the maximum daily rate of nitrogen accumulation (MXNUP) ranged from −0.3 % to 1.9 %. The SNCG, SNCS, and SLNS cultivars exhibited a statistically significant increase in yield compared to the STD cultivars (except for SNCS in Gorgan), ranging from 0.2 % to 1.9 %. However, the SLNG and MXNUP cultivars did not show any significant yield increase in any of the studied zones.

## 4 Discussion

### 4.1 Modified cultivars

**Fig. 4:**
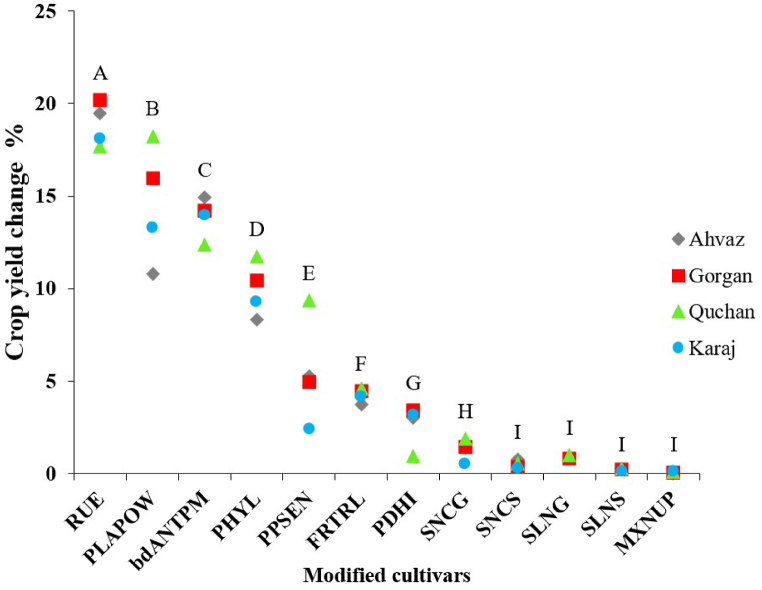
Percentage of crop yield change in modified cultivars. Letters compare differences of the cultivars over the selected environments; cultivars with the same letter have no significant difference at P = 0.05 with respect to the percentage change in crop yield.

The effectiveness of the evaluated traits across the environments was varied (Fig. 4). Among these traits, increasing RUE emerged as the most promising, leading to an average crop yield increase of 18.9 % (ranging from 18 to 20 %). Stella et al. [17] also observed that RUE had the most significant marginal effect on yield under irrigated conditions. Enhancing RUE has long been a focal point for plant physiologists and breeders aiming to improve crop yield [47]. Traditionally, it was believed that RUE could not be substantially increased through conventional breeding methods [16,48]. However, Reynolds et al. [49] emphasized the importance of this trait and reviewed physiological strategies to enhance RUE in wheat. They suggested that by combining information on genetic limitations to crop photosynthetic rate with new physiological selection tools, the probability of improving RUE through plant breeding could be significantly increased. Newly [50], proposed targeting RUE by measuring low-light photosynthetic performance, simplifying the laborious RUE phenotyping process and opening new possibilities for utilizing high-throughput phenotyping methodologies to screen for improved crop RUE. The findings of the present study confirm the substantial impact of RUE on yield enhancement, underscoring the need for further research efforts to uncover methods for enhancing this trait. In various studies, the RUE of wheat genotypes has been reported to range from 1.11 to 3 g MJ^−1^ [40,51,52].

The second most effective trait was faster leaf area expansion (PLAPOW), which resulted in an 11 %–18 % increase in yield (average = 15 %). Soltani and Galeshi [15] reported a 7 %–10 % yield increase in wheat due to accelerated leaf area development resulting from a 20 % increase in the relative expansion rate of the leaf area index. They also found significant genetic diversity for this trait among the 13 wheat cultivars studied. In addition [53], assessed the benefits of early vigor (faster early leaf area development) in wheat in dryland regions of Australia and concluded that there are opportunities for developing early vigor wheat varieties for wetter sites.

Increasing the grain filling period (bdANTPM) ranked as the third most effective trait, leading to a 12 %–15 % increase in crop yield (average = 14 %). These results are consistent with other similar studies [54]. While grain filling is a complex physiological process, several major quantitative trait loci related to it have been discovered, providing breeders with the means to increase the grain filling rate through targeted breeding programs [55,56]. Notably, significant genotypic variations (ranging from 500 to 840-degree days) have been observed for the length of the grain filling period among wheat genotypes [16,55,57]. However, it is important to consider the implications of modifying the growth period of a crop plant, as it may result in the loss of the optimal sowing window for the subsequent crop after wheat, such as in wheat-soybean double cropping [58].

Faster leaf production through a smaller phyllochron occupied the fourth position, resulting in an average yield increase of 10 %, ranging between 8 % and 12 % depending on the environment. Various studies have reported genetic diversity in the phyllochron of wheat genotypes, ranging from 70 to 130 °C per leaf, indicating a high diversity of this trait [39,59,60]. This substantial genotypic diversity makes phyllochron a suitable trait for improvement [61,62].

The fifth most effective trait was longer vegetative growth through photoperiod sensitivity (PPSEN), resulting in a yield increase of 2 %–9 % (average = 5.5 %). The response to photoperiod is one of the main determinants of the rate of phenological development of wheat in a specific region, regulated by various alleles [63]. Wheat possesses a large number of genes and alleles (Vrn-1 for vernalization, Ppd-1 for photoperiod, Eps for early maturity) that influence development rate, their interactions, and different sensitivities in different growth stages [64]. This indicates the possibility of achieving a longer growth period (delay in flowering) through breeding. The variation in photoperiod sensitivity coefficient among wheat genotypes is substantial, with a wide range of PPSEN observed in various studies [43,65,66]. Previously, the genetic diversity of alleles affecting photoperiod sensitivity and agricultural practices, such as changing planting dates, has been used to regulate the growth period of wheat [64,67].

The effects of the traits FRTRL and PDHI were 4 % and 3 %, respectively. Various studies have reported FRTRL ranging from 0.19 to 0.28 g g^−1^ in wheat genotypes [7,19,41]. Although there is genetic variability for this trait, successful improvement in breeding programs based on FRTRL has not been observed. Increasing the slope of linear increase in harvest index (PDHI) can lead to an increase in seed growth rate and also the rate of nitrogen translocation from leaves and stems to grains. However, this may accelerate leaf senescence, resulting in decreased crop dry matter production during the grain-filling period, potentially negatively impacting yield. Therefore, in the current study, increasing this trait did not have a considerable impact on yield. The range of PDHI in wheat genotypes has been reported as 0.011–0.02 g g^−1^ in various reports [19,26,41,68]. Different studies have reported changes in the harvest index ranging from 0.22 to 0.62 in bread wheat cultivars [[69], [70], [71]]. In a study of 30 bread wheat genotypes, a high genetic heritability (61 %) and genetic advance (16 %) were reported for the harvest index [72]. High genetic heritability for the harvest index in wheat has also been reported in other studies [73,74].

Among the nitrogen-related traits (SLNG, SLNS, SNCG, SNCS, and MXNUP cultivars) and across the selected locations for the study, only SNCG showed a significant effect on crop yield (average = 1.3 %), while the effect of other nitrogen-related traits was not significantly different from zero (Fig. 4). These results were unexpected, as modifying these traits was expected to increase crop yield through enhanced nitrogen accumulation and/or translocation from leaves and stems to grains. Nitrogen accumulation by the crop can be calculated as the product of dry matter accumulation and nitrogen concentration. Among the nitrogen-related traits evaluated, only SLNG (6–10 %) and SNCG (7–10 %) had a significant impact on nitrogen accumulation, while the hypothetical cultivars SLNS, SNCS, and MXNUP did not show any significant effect on nitrogen accumulation compared to the STD cultivars (data shown in Table S3 in SI). However, N accumulation in the hypothetical cultivars RUE and PLAPOW was significantly higher than in the STD cultivars (Table S3 in SI) due to their effect on dry matter accumulation. Another factor contributing to the limited impact of nitrogen-related traits is the implementation of automatic nitrogen fertilization, which created optimal nitrogen conditions for plant growth. Experimental data have indicated that increasing nitrogen concentration under such conditions has no effect on crop yield [[75], [76], [77], [78]].

Different wheat cultivars exhibit a wide range of specific nitrogen content in green leaves (SLNG), ranging from 1.4 to 7.16 g m^−2^ [46,68]. The specific nitrogen content of yellow leaves (SLNS) has been reported to range from 0.1 to 1.28 g m^−2^ in various studies [41,46]. Nitrogen concentration in young wheat stems (SNCG) has been reported to range from 0.006 to 0.027 g g^−1^ in different wheat cultivars [45,46,79], while in senescent stems (SNCS), the range is from 0.003 to 0.021 g g^−1^ [46,80,81]. The accumulated nitrogen content in the aerial part at the physiological maturity stage varies significantly among wheat cultivars. For example, a study of seven wheat cultivars reported this trait to range from 15.4 to 21.5 g m^−2^ [80], while another study of 30 wheat cultivars reported a range of 10.3–19.8 g m^−2^ with a coefficient of variation of 30 % [82].

Overall, improving RUE offers the greatest potential for increasing wheat yield under irrigated conditions. However, improving RUE remains challenging, and there is a need to find and validate simple measurements and phenotyping methods targeting RUE [50]. Breeding longer duration cultivars (bdANTPM and PPSEN) may not be practical in all regions, as late-maturing cultivars can disrupt cropping systems by interfering with the optimal sowing window for the next crop. Therefore, in the short term, the most significant yield increase under optimal irrigated conditions can be achieved by breeding for faster leaf area development (PLAPOW and PHYL). Fortunately, methods are available for phenotyping traits related to leaf development (e.g. Ref. [83]), allowing for high-rate and precise phenotyping of these traits.

### **4.2** Increased nitrogen and water uptake are essential for higher yields

**Fig. 5:**
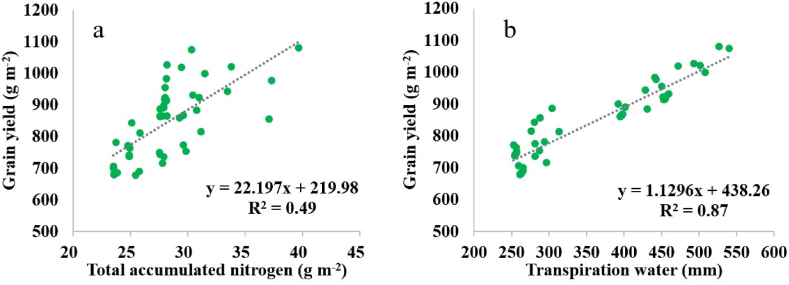
Grain yield versus (a) accumulated nitrogen in the above-ground crop organs and (b) total plant transpiration. Each point is the 15-year average yield of the standard cultivar or a modified cultivar in the study zones.

The increased yield in the modified cultivars in the current study was strongly associated with nitrogen and water uptake, as depicted in Fig. 5. Limited access to two crucial resources, water and nitrogen, often severely restricts crop growth and yield [18]. Therefore, genetic improvement of crops is unlikely to be successful without enhancing nitrogen accumulation and water uptake by the crop [18]. The study revealed that the crop yield increased by 22 g m^−2^ for every g m^−2^ of nitrogen uptake, indicating that the hypothetical cultivars exhibited increased yield due to enhanced nitrogen uptake. Compared to the STD cultivars (260 kg N ha^−1^), the RUE cultivar required 9 % more nitrogen accumulation (283 kg N ha^−1^), the PLAPOW cultivar required 38 % more (360 kg N ha^−1^), the PHYL cultivar required 21 % more (316 kg N ha^−1^), and the PPSEN cultivar required 15 % more (299 kg N ha^−1^) (Fig. 5a). Other studies have also reported a positive and significant relationship between yield and nitrogen accumulation [31,68]. Sinclair and Rufty [18] demonstrated that in most cases, yield formation is limited by nitrogen accumulation, and achieving high yields is more likely when nitrogen accumulation is also high. They emphasized that increasing nitrogen storage in the vegetative organs of the crop was the key solution to increasing crop yield. Furthermore, several studies have shown that dry matter and nitrogen accumulation exhibit common genetic correlations [55].

The process of photosynthesis and plant growth requires carbon dioxide (CO_2_), and to facilitate this, plants keep their stomata open during specific periods of the day, which results in transpiration. This relationship is quantitatively defined by the physics of gas diffusion and often imposes significant limitations on yield [18]. The results of the current study demonstrated a strong linear correlation (R^2^ = 0.87) between yield in the modified cultivars and transpiration. Specifically, for every 1 mm of transpired water, the yield increased by 1.1 g m^−2^ (Fig. 5b).

## 5 Conclusions

The results of this study demonstrated that modifying the current cultivars to enhance RUE, accelerate leaf area expansion (PHYL and PLAPOW), and extend the grain filling and vegetative periods led to a significant improvement in yield by more than 5 %, across various irrigated wheat growing environments in Iran. However, the specific impact of these traits on yield varied slightly in different climatic zones. Considering the challenges associated with breeding for RUE and the potential disruption of cropping systems caused by longer duration cultivars, it was concluded that, in the short term, breeding for faster leaf area development and rapid canopy closure would be the most promising approach to increase irrigated wheat yield under optimal water and nitrogen management. Additionally, there is potential for further yield improvement by combining these promising traits, which warrants additional research efforts. It is important to note that the modified cultivars would require increased water and nitrogen inputs to support higher grain yields. These research findings have significant implications for the development of more productive wheat cultivars, which are crucial in ensuring food security.

## Data/code availability statement

The datasets and codes generated during and/or analyzed during the current study are available from the corresponding authors on reasonable request.

## CRediT authorship contribution statement

**Arezoo Abidi:** Writing – original draft, Investigation, Methodology, Writing – review & editing. **Afshin Soltani:** Writing – review & editing, Supervision, Conceptualization, Methodology, Resources, Writing – original draft. **Ebrahim Zeinali:** Writing – review & editing, Supervision, Methodology, Resources.

## Declaration of competing interest

The authors declare that they have no known competing financial interests or personal relationships that could have appeared to influence the work reported in this paper.

## References

[1] Alexandratos N., Bruinsma J. (2012).

[2] Cassman K.G. (2012). What do we need to know about global food security?. Global Food Secur..

[3] Senapati N., Semenov M.A. (2020). Large genetic yield potential and genetic yield gap estimated for wheat in Europe. Global Food Secur..

[4] Van Ittersum M.K., Cassman K.G., Grassini P., Wolf J., Tittonell P., Hochman Z. (2013). Yield gap analysis with local to global relevance-A review. Field Crops Res..

[5] Asseng S., Martre P., Ewert F., Dreccer M.F., Beres B.L., Reynolds M., Braun H.J., Langridge P., Le Gouis J., Salse J., Baenziger P.S. (2019). Model-driven multidisciplinary global research to meet future needs: the case for “improving radiation use efficiency to increase yield,”. Crop Sci..

[6] Jordan W.R., Dugas W.A., Shouse P.J. (1983). Strategies for crop improvement for drought-prone regions. Agric. Water Manag..

[7] Soltani A., Sinclair T.R. (2012).

[8] Sinclair T.R. (2011). Challenges in breeding for yield increase for drought. Trends Plant Sci..

[9] Sinclair T.R., Soltani A., Marrou H., Ghanem M., Vadez V. (2019). Geospatial assessment for crop physiological and management improvements with examples using the simple simulation model. Crop Sci..

[10] Tao F., Rötter R.P., Palosuo T., Díaz-Ambrona C.G.H., Mínguez M.I., Semenov M.A., Kersebaum K.C., Nendel C., Cammarano D., Hoffmann H., Ewert F., Dambreville A., Martre P., Rodríguez L., Ruiz-Ramos M., Gaiser T., Höhn J.G., Salo T., Ferrise R., Bindi M., Schulman A.H. (2017). Designing future barley ideotypes using a crop model ensemble. Eur. J. Agron..

[11] Muller B., Martre P. (2019). Plant and crop simulation models: powerful tools to link physiology, genetics, and phenomics. J. Exp. Bot..

[12] Sciarresi C., Patrignani A., Soltani A., Sinclair T., Lollato R.P. (2019). Plant traits to increase winter wheat yield in semiarid and subhumid environments. Agron. J..

[13] Senapati N., Stratonovitch P., Paul M.J., Semenov M.A. (2019). Drought tolerance during reproductive development is important for increasing wheat yield potential under climate change in Europe. J. Exp. Bot..

[14] Sadok W., Schoppach R., Ghanem M.E., Zucca C., Sinclair T.R. (2019). Wheat drought-tolerance to enhance food security in Tunisia, birthplace of the Arab Spring. Eur. J. Agron..

[15] Soltani A., Galeshi S. (2002). Importance of rapid canopy closure for wheat production in a temperate sub-humid environment: experimentation and simulation. Field Crops Res..

[16] Asseng S., Turner N.C., Ray J.D., Keating B.A. (2002). A simulation analysis that predicts the influence of physiological traits on the potential yield of wheat. Eur. J. Agron..

[17] Stella T., Webber H., Rezaei E.E., Asseng S., Martre P., Dueri S., Guarin J.R., Pequeno D.N.L., Calderini D.F., Reynolds M., Molero G., Miralles D., Garcia G., Slafer G., Giunta F., Kim Y.U., Wang C., Ruane A.C., Ewert F. (2023).

[18] Sinclair T.R., Rufty T.W. (2012). Nitrogen and water resources commonly limit crop yield increases, not necessarily plant genetics. Global Food Secur..

[19] Soltani A., Maddah V., Sinclair T.R. (2013). SSM-wheat: a simulation model for wheat development, growth and yield. Int. J. Plant Prod..

[20] Soltani A., Alimagham S.M., Nehbandani A., Torabi B., Zeinali E., Dadrasi A., Zand E., Ghassemi S., Pourshirazi S., Alasti O., Hosseini R.S., Zahed M., Arabameri R., Mohammadzadeh Z., Rahban S., Kamari H., Fayazi H., Mohammadi S., Keramat S., Vadez V., van Ittersum M.K., Sinclair T.R. (2020). SSM-iCrop2: a simple model for diverse crop species over large areas. Agric. Syst..

[21] Schoppach R., Sinclair T.R., Sadok W. (2020). Sleep tight and wake-up early: nocturnal transpiration traits to increase wheat drought tolerance in a Mediterranean environment. Funct. Plant Biol..

[22] Messina C.D., Sinclair T.R., Hammer G.L., Curan D., Thompson J., Oler Z., Gho C., Cooper M. (2015). Limited-transpiration trait may increase maize drought tolerance in the US corn belt. Agron. J..

[23] Sinclair T.R., Messina C.D., Beatty A., Samples M. (2010). Assessment across the United States of the benefits of altered soybean drought traits. Agron. J..

[24] Zahed M., Soltani A., Zeinali E., Torabi B., Zand E. (2018). Gorgan University of Agricultural Sciences and Natural Resources.

[25] Van Wart J., Kersebaum K.C., Peng S., Milner M., Cassman K.G. (2013). Estimating crop yield potential at regional to national scales. Field Crops Res..

[26] Amir J., Sinclair T.R. (1991). A model of the temperature and solar-radiation effects on spring wheat growth and yield. Field Crops Res..

[27] Amir J., Sinclair T.R. (1991). A model of water limitation on spring wheat growth and yield. Field Crops Res..

[28] Sinclair T.R., Amir J. (1992). A model to assess nitrogen limitations on the growth and yield of spring wheat. Field Crops Res..

[29] Soltani A., Sinclair T.R. (2011). A simple model for chickpea development, growth and yield. Field Crops Res..

[30] Soltani A., Sinclair T.R. (2015). A comparison of four wheat models with respect to robustness and transparency: simulation in a temperate, sub-humid environment. Field Crops Res..

[31] Soltani A., Sinclair T.R., Zeinali E., Abidi A. (2022). Assessing plant nitrogen-use dynamics traits for increasing wheat mass accumulation and grain yield. Gorgan Univ. Agric. Sci. Nat. Resour..

[32] Lollato R.P., Edwards J.T., Ochsner T.E. (2017). Meteorological limits to winter wheat productivity in the U.S. southern Great Plains. Field Crops Res..

[33] Shaaban A.S.A., Wahbi A., Sinclair T.R. (2018). Sowing date and mulch to improve water use and yield of wheat and barley in the Middle East environment. Agric. Syst..

[34] Schoppach R., Soltani A., Sinclair T.R., Sadok W. (2017). Yield comparison of simulated rainfed wheat and barley across Middle-East. Agric. Syst..

[35] Bregaglio S., Willocquet L., Kersebaum K.C., Ferrise R., Stella T., Ferreira T.B., Pavan W., Asseng S., Savary S. (2021). Comparing process-based wheat growth models in their simulation of yield losses caused by plant diseases. Field Crops Res..

[36] Manschadi A.M., Palka M., Fuchs W., Neubauer T., Eitzinger J., Oberforster M., Soltani A. (2022). Performance of the SSM-iCrop model for predicting growth and nitrogen dynamics in winter wheat. Eur. J. Agron..

[37] Koo J., Dimes J. (2013).

[38] Nehbandani A., Soltani A., Taghdisi Naghab R., Dadrasi A., Alimagham S.M. (2020). Assessing HC27 soil database for modeling plant production. Int. J. Plant Prod..

[39] Alderman P., Quilligan E., Asseng S., Ewert F., Reynolds M. (2013).

[40] Yunusa I.A.M., Siddique K.H.M., Belford R.K., Karimi M.M. (1993). Effect of canopy structure on efficiency of radiation interception and use in spring wheat cultivars during the pre-anthesis period in a mediterranean-type environment. Field Crops Res..

[41] Fuchs W., Manschadi A.M. (2021).

[42] Austin R.B., Bingham J., Blackwell R.D., Evans L.T., Ford M.A., Morgan C.L., Taylor M. (1980). Genetic improvements in winter wheat yields since 1900 and associated physiological changes. J. Agric. Sci., Camb..

[43] Panahi M.H., Soltani A., Zeinali E., Kalateh Arabi M., Nehbandani A.R. (2019). Estimation of phenological parameters in SSM-Wheat model for bread wheat (Triticum aestivum L.) genotypes in Golestan province of Iran., Iran. J. Crop Sci..

[44] Alipour H., Abdi H., Bihamta M.R. (2020). Assessment of growing degree-days values of phenological stages in some Iranian bread wheat cultivars and landraces. J. Crop Breed..

[45] Bayat Z., Ahmadi A., Sabokdast M. (2011). Evaluation of genotypic variety for grain yield and protein and its relationship with nitrogen remobilization in Iranian wheat cultivars. Iran. J. Field Crop Sci..

[46] Soltani A., Nehbandani A.R., Zeinali E. (2017).

[47] Reynolds M., Bonnett D., Chapman S.C., Furbank R.T., Manés Y., Mather D.E., Parry M.A.J. (2011). Raising yield potential of wheat. I. Overview of a consortium approach and breeding strategies. J. Exp. Bot..

[48] Sinclair T.R., Keating B.A., Wilson J.R. (1996). Intensive Sugarcane Production: Meeting the Challenges beyond 2000. Proceedings of the Sugar 2000 Symposium. Brisbane, Australia.

[49] Reynolds M.P., Van Ginkel M., Ribaut J.M. (2000). Avenues for genetic modification of radiation use efficiency in wheat. J. Exp. Bot..

[50] Wu A., Huynh Truong S., McCormick R., Van Oosterom E.J., Messina C.D., Cooper M., Hammer G.L. (2024). Contrasting leaf-scale photosynthetic low-light response and its temperature dependency are key to differences in crop-scale radiation use efficiency. New Phytol..

[51] Dastmalchi A., Soltani A., Latifi N., Zeinali E. (2011). Parameter estimates and evaluation of CropSyst-Wheat for Golestan province cultivars. EJCP.

[52] Shearman V.J., Sylvester-Bradley R., Scott R.K., Foulkes M.J. (2005). Physiological processes associated with wheat yield progress in the UK. Crop Sci..

[53] Zhao Z., Rebetzke G.J., Zheng B., Chapman S.C., Wang E. (2019). Modelling impact of early vigour on wheat yield in dryland regions. J. Exp. Bot..

[54] Monpara B.A. (2011). Grain filling period as a measure of yield improvement in bread wheat. Crop Improv..

[55] Charmet G., Robert N., Branlard G., Linossier L., Martre P., Triboï E. (2005). Genetic analysis of dry matter and nitrogen accumulation and protein composition in wheat kernels. Theor. Appl. Genet..

[56] Wang R.X., Hai L., Zhang X.Y., You G.X., Yan C.S., Xiao S.H. (2009). QTL mapping for grain filling rate and yield-related traits in RILs of the Chinese winter wheat population Heshangmai x Yu8679. Theor. Appl. Genet..

[57] Akkaya A., Dokuyucu T., Kara R., Akçura M. (2006). Harmonization ratio of post- to pre-anthesis durations by thermal times for durum wheat cultivars in a Mediterranean environment. Eur. J. Agron..

[58] Parvej M.R., Holshouser D.L., Kratochvil R.J., Whaley C.M., Dunphy E.J., Roth G.W., Faé G.S. (2020). Early high-moisture wheat harvest improves double-crop system: II. Soybean growth and yield. Crop Sci..

[59] Mosaad M.G., Ortiz-Ferrara G., Mahalakshmi V., Fischer R.A. (1995). Phyllochron response to vernalization and photoperiod in spring wheat. Crop Sci..

[60] Fuchs W. (2016).

[61] Hay R.K.M., Kirby E.J.M. (1991). Convergence and synchrony-a review of the coordination of development in wheat. Aust. J. Agric. Res..

[62] He J., Le Gouis J., Stratonovitch P., Allard V., Gaju O., Heumez E., Orford S., Griffiths S., Snape J.W., Foulkes M.J., Semenov M.A., Martre P. (2012). Simulation of environmental and genotypic variations of final leaf number and anthesis date for wheat. Eur. J. Agron..

[63] Whittal A., Kaviani M., Graf R., Humphreys G., Navabi A. (2018). Allelic variation of vernalization and photoperiod response genes in a diverse set of North American high latitude winter wheat genotypes. PLoS One.

[64] Snape J.W., Butterworth K., Whitechurch E., Worland A.J. (2001). Waiting for fine times: genetics of flowering time in wheat. Euphytica.

[65] Bloomfield M.T., Celestina C., Hunt J.R., Huth N., Zheng B., Brown H., Zhao Z., Wang E., Stefanova K., Hyles J., Rathjen T., Trevaskis B. (2023). Vernalisation and photoperiod responses of diverse wheat genotypes. Crop Pasture Sci..

[66] Celestina C., Hunt J., Kuchel H., Harris F., Porker K., Biddulph B., Bloomfield M., McCallum M., Graham R., Matthews P., Aisthorpe D., Al-Yaseri G., Hyles J., Trevaskis B., Wang E., Zhao Z., Zheng B., Huth N., Brown H. (2023). A cultivar phenology classification scheme for wheat and barley. Eur. J. Agron..

[67] Worland A.J., Korzun V., Röder M.S., Ganal M.W., Law C.N. (1998). Genetic analysis of the dwarfing gene Rht8 in wheat. Part II. The distribution and adaptive significance of allelic variants at the Rht8 locus of wheat as revealed by microsatellite screening. Theor. Appl. Genet..

[68] Manschadi A.M., Soltani A. (2021). Variation in traits contributing to improved use of nitrogen in wheat: implications for genotype by environment interaction. Field Crops Res..

[69] Khalilzadeh G., Arshad Y., Rezayi M., Eyvazi A. (2012). Evaluation of yield, yield components, uptake and use efficiency of nitrogen in wheat cultivars (Triticum aestivum L.). J. Reasearch Crop Sci..

[70] Barani S., Shokrpour M. (2013). Evaluation of some agricultural and phenological traits in different genotypes of spring bread wheat. J. New Find. Agric..

[71] Wnuk A., Górny A.G., Bocianowski J., Kozak M. (2013). Visualizing harvest index in crops, Commun. Biometry Crop Sci..

[72] Parveen R., Kumar Singh S., Jaiswal P., kumar Singh M., Barman M. (2021). Genetic variability analysis in bread wheat (Triticum aestivum L.) genotypes for early heat tolerance and grain zinc content. Pharm. Innov..

[73] Ajmal S.U., Zakir N., Mujahid M.Y. (2009). Estimation of genetic parameters and character association in wheat. J. Agric. Biol. Sci..

[74] Mangi S.A., Sial M.A., Ansari B.A., Afzal Arain M., Laghari K.A., Mirbahar A.A. (2010). Heritability studies for grain yield and yield components in F3 segregating generation of spring wheat. Pakistan J. Bot..

[75] Ma G., Liu W., Li S., Zhang P., Wang C., Lu H., Wang L., Xie Y., Ma D., Kang G. (2019). Determining the optimal N input to improve grain yield and quality in winter wheat with reduced apparent N loss in the north China plain. Front. Plant Sci..

[76] Chen Y., Xiao C., Wu D., Xia T., Chen Q., Chen F., Yuan L., Mi G. (2015). Effects of nitrogen application rate on grain yield and grain nitrogen concentration in two maize hybrids with contrasting nitrogen remobilization efficiency. Eur. J. Agron..

[77] Lemaire G., Jeuffroy M.H., Gastal F. (2008). Diagnosis tool for plant and crop N status in vegetative stage. Theory and practices for crop N management. Eur. J. Agron..

[78] Ziadi N., Bélanger G., Claessens A., Lefebvre L., Cambouris A.N., Tremblay N., Nolin M.C., Parent L.É. (2010). Determination of a critical nitrogen dilution curve for spring wheat. Agron. J..

[79] Triboi E., Ollier J.L. (1991). Evolution et rôle des réserves glucidiques et azotées des tiges chez 21 génotypes de blé. Agronomie.

[80] Bakhshandeh E., Soltani A., Zeinali E., Ghadiryan R. (2013). Study of dry matter and nitrogen accumulation, remobilization and harvest index in bread and durum wheat cultivars. EJCP.

[81] Kim N.I., Paulsen G.M. (1986). Assimilation and partitioning of photosynthate and nitrogen in isogenic tall, semidwarf, and doubledwarf winter wheats. J. Agron. Crop Sci..

[82] Nehe A.S., Misra S., Murchie E.H., Chinnathambi K., Foulkes M.J. (2018). Genetic variation in N-use efficiency and associated traits in Indian wheat cultivars. Field Crops Res..

[83] Vadez V., Kholová J., Hummel G., Zhokhavets U., Gupta S.K., Hash C.T. (2015). LeasyScan: a novel concept combining 3D imaging and lysimetry for high-throughput phenotyping of traits controlling plant water budget. J. Exp. Bot..

