# Supplementary material for: Identifying plant traits to increase wheat yield under irrigated conditions
Source: Heliyon. 2024 May 22;10(11):e31734. doi: 10.1016/j.heliyon.2024.e31734 (PMC11154629; doi:10.1016/j.heliyon.2024.e31734)
Supplement: Multimedia component 1 [file mmc1.pdf]

Supplementary Information

Identifying plant traits to increase wheat yield under irrigated conditions

Arezoo Abidi *, Afshin Soltani *, Ebrahim Zeinali

Department of Agronomy, Gorgan University of Agricultural Sciences and Natural Resources, Gorgan, 4918943464, Iran

* Corresponding author.

E-mail addresses: arezooabidi@gmail.com (A. Abidi), afshin.soltani@gmail.com (A. Soltani)

Table S1. Soil input parameters as used in the SSM-iCrop model for the four studied zones. DLYER: layer thickness (mm), SAT: saturated water content (cm^3 cm^{-3}), DUL: drained upper limit (cm^3 cm^{-3}), LL: lower limit (cm^3 cm^{-3}), ADRY: air-dry soil water content (cm^3 cm^{-3}), iniWL: initial soil water (cm^3 cm^{-3}), DRAINF: drainage factor, FG: fraction coarse material in the soil (%), BDL: bulk density (g cm^{-3}), NORG: soil organic N (%), FMIN: fraction of soil organic N available for mineralization (%), iNSOL: initial soil nitrogen in soil solution (kg N ha^{-1}), SALB: Albedo, CN2: Curve number

		**Ahvaz**												
**Layer**	**DLYER**	**SAT**	**DUL**	**LL**	**ADRY**	**iniWL**	**DRAINF**	**FG**	**BDL**	**NORG**	**FMIN**	**iNSOL**	**SALB**	**CN2**
1	100	0.4	0.301	0.17	0.057	0.27	0.5	0	1.4	0.04	0.15	2.56	0.1	75
2	200	0.41	0.31	0.18	0.06	0.30	0.5	0	1.4	0.03	0.11	4.11		
3	300	0.42	0.31	0.19	0.19	0.30	0.5	0	1.4	0.02	0.08	5.62		
4	300	0.43	0.315	0.215	0.215	0.31	0.5	0	1.4	0.02	0.06	5.39		
5	300	0.44	0.317	0.25	0.25	0.31	0.5	0	1.4	0.02	0.05	5.28		
		**Gorgan, Quchan and Karaj**												
**Layer**	**DLYER**	**SAT**	**DUL**	**LL**	**ADRY**	**iniWL**	**DRAINF**	**FG**	**BDL**	**NORG**	**FMIN**	**iNSOL**	**SALB**	**CN2**
1	100	0.44	0.39	0.21	0.07	0.35	0.2	0	1.2	0.10	0.15	3.33	0.05	85
2	200	0.467	0.41	0.23	0.08	0.39	0.2	0	1.2	0.06	0.10	5.43		
3	300	0.467	0.415	0.26	0.26	0.40	0.2	0	1.2	0.05	0.06	7.26		
4	300	0.47	0.42	0.29	0.29	0.41	0.2	0	1.2	0.05	0.03	6.96		
5	300	0.475	0.425	0.32	0.32	0.41	0.2	0	1.2	0.04	0.02	6.81		

Table S2. Definition of crop parameters in the SSM-iCrop model for wheat and their estimates for current cultivars. STD1 is typical cultivar in regions with moderate winters and STD2 is typical cultivar in regions with cold winters.

Parameter	Abbreviation	STD1	STD2
Phenology			
Base temperature for development (°C)	TBD	0	0
Optimum temperature for development (°C)	TP1D	27.5	27.5
Ceiling temperature for development (°C)	TCD	40	40
Vernalization sensitivity coefficient	vsen	0.00089	0.0015
Critical photoperiod (h)	cpp	21	21
Photoperiod sensitivity coefficient	ppsen	0.001467	0.001467
Biological days from sowing to emergence (Biological day)	bdSOWEMR	3.5	3.5
Biological days from emergence to first-tiller (Biological day)	bdEMRTIL	5	5
Biological days from first-tiller to first-node (stem-elongation) (Biological day)	bdTILSEL	11.5	11.5
Biological days from first-node to booting (ligule of flag leaf visible) (Biological day)	bdSELBOT	7	7
Biological days from booting to ear emergence (Biological day)	bdBOTEAR	3	3
Biological days from ear emergence to anthesis (Biological day)	bdEARANT	8	8
Biological days from anthesis to physiological maturity (Biological day)	bdANTPM	34	34
Biological days from physiological maturity to harvest maturity (Biological day)	bdPMHM	8	8
Leaf area development and senescence			
Phyllochron, the accumulated thermal time required for the successive leaves' appearance in the main stem (°C $leaf^{-1}$)	Phyl	95	95
A coefficient (constant) in the power relationship between plant leaf area and mainstem node number	PLACON	1	1
A coefficient (exponent) in the power relationship between plant leaf area and main stem node number (For 300 plants m^{-2})	PLAPOW	2.4336	2.4336
A coefficient (constant) in the relationship between PLAPOW and plant density	a_plapow d	1.1718	1.1718
A coefficient (constant) in the relationship between PLAPOW and plant density	b_plapow_d	-0.0006	-0.0006
Specific leaf area (m^2 g^{-1})	SLA	0.021	0.021
Low temperature/freezing threshold for leaf death (°C)	FrzTh	-5	-5
Fraction leaf destruction below the critical by each degree centigrade (m^2 m^{-2})	FrzLDR	0.01	0.01
Heat threshold temperature for leaf senescence (°C)	HeatTH	30	30
Relative increase in leaf senescence rate per each degree above heat threshold (°C)	HtLDR	0.1	0.1
Dry mass accumulation			
Base temperature for dry matter production (°C)	TBRUE	0	0
Lower optimum temperature for dry matter production (°C)	TP1RUE	15	15
Upper optimum temperature for dry matter production (°C)	TP2RUE	22	22
Ceiling temperature for dry matter production (°C)	TCRUE	35	35
Extinction coefficient for photosynthetically active radiation	KPAR	0.65	0.65
Radiation use efficiency under optimal growth conditions (g MJ^{-1})	IRUE	2.42	2.42
A coefficient that describes RUE response to CO_2 in a curvinial response.	CO2RES	0.8	0.8
Dry mass partitioning			
Partitioning coefficient to leaves during the main phase of leaf area development at lower levels of total crop mass (g g^{-1})	FLF1A	0.6	0.6
Partitioning coefficient to leaves during the main phase of leaf area development at higher levels of total crop mass (g g^{-1})	FLF1B	0.3	0.3
Total crop mass when leaf partitioning coefficient turns from FLF1A to FLF1B (g g^{-1})	WTOPL	160	160
Partitioning coefficient to leaves from termination leaf growth on mainstem to beginning seed growth (g g^{-1})	FLF2	0.1	0.1
Yield formation			
Fraction crop mass at the beginning of seed growth which is translocatable to grains (g g^{-1})	FRTRL	0.22	0.22
Grain conversion coefficient (g g^{-1})	GCF	1	1
The rate of linear increase in harvest index during effective grain filling period (g g^{-1} d^{-1})	PDHI	0.019	0.019
A turning point in relationship between PDHI and crop dry mass at beginning grain filling (g m^{-2})	WDHI1	0	0
A turning point in relationship between PDHI and crop dry mass at beginning grain filling (g m^{-2})	WDHI2	600	600
A turning point in relationship between PDHI and crop dry mass at beginning grain filling (g m^{-2})	WDHI3	1200	1200
A turning point in relationship between PDHI and crop dry mass at beginning grain filling (g m^{-2})	WDHI4	3200	3200
Critical high temperature for yield formation via PHDI (°C)	TP2H	31	31
Ceiling (maximum) temperature for yield formation via PHDI (°C)	TCH	40	40
Critical low temperature for yield formation via PHDI (°C)	TP1F	0	0

Ceiling (minimum) temperature for yield formation via PHDI (°C)	TBF	-5	-5
The maximum fraction of flowers that open on a day	Rfmax	0.3	0.3
Water relations			
Initial depth of roots at emergence or beginning leaf growth (mm)	iDEPORT	200	200
Maximum effective depth of water extraction from soil (mm)	MEED	1000	1000
Potential daily increase (growth) in root depth (mm d^{-1})	GRTDP	30	30
Transpiration efficiency coefficient (Pa)	TEC	5.8	5.8
FTSW* threshold when dry matter production starts to decline	WSSG	0.3	0.3
FTSW threshold when leaf area development starts to decline	WSSL	0.4	0.4
A coefficient that specifies acceleration or retardation in development in response to water deficit	WSSD	0.5	0.5
Killing number of consecutive flooding (day)	FLDKL	99	99
Plant nitrogen budget			
Specific leaf nitrogen in green leaves (target) (g N m^{-2})	SLNG	1.8	1.8
Specific leaf nitrogen in senesced leaves (minimum) (g N m^{-2})	SLNS	0.4	0.4
Stem nitrogen concentration in green stems (target) (g N g^{-1})	SNCG	0.019	0.019
Stem nitrogen concentration in senesced stems before beginning of seed growth (minimum) (g N g^{-1})	SNCS1	0.0095	0.0095
Stem nitrogen concentration in senesced stems after beginning of seed growth (minimum) (g N g^{-1})	SNCS2	0.0022	0.0022
Grain nitrogen concentration (minimum)	GNCmin	0.016	0.016
Grain nitrogen concentration (maximum)	GNCmax	0.035	0.035
Maximum rate of nitrogen uptake (g N m^{-2} d^{-1})	MXNUP	0.6	0.6

*FTSW: fraction transpirable soil water

Table S3. Days to anthesis (DtANT), days to harvest maturity (DtHAR), maximum LAI (MXLAI), total dry matter (WTOP), harvest index (HI), grain dry matter (WGRN), cumulative intercepted PAR (intPAR), total nitrogen accumulation in crop above-ground organs (NUP), total applied N fertilizer (NFERT), nitrogen leached from the soil (NLEACH), Cumulative transpiration (CTR) and Cumulative irrigation water (CIRGW) for standard and modified cultivars in the four study environments

	Ahvaz											
	**DtANT**	**DtHAR**	**MXLAI**	**WTOP**	**HI**	**WGRN**	**intPAR**	**NUP**	**NFERT**	**NLEACH**	**CTR**	**CIRGW**
**Cultivar**	(d)	(d)	(m^2 m^{-2})	(g m^{-2})	(g g^{-1})	(g m^{-2})	(MJ m^{-2})	(g N m^{-2})	(g N m^{-2})	(g N m^{-2})	(mm)	(mm)
**STD**	84.3^{a}	137.5^{b}	6.39^{c}	1300.0^{b}	0.52^{b}	679.9^{c}	608.6^{b}	23.6^{cde}	17.7^{d}	0.45^{a}	262.6^{d}	332.0^{b}
**PHYL**	84.3^{a}	137.5^{b}	8.70^{ab}	1390.1^{b}	0.53^{b}	736.5^{abc}	655.7^{b}	27.9^{ab}	22.3^{ab}	0.39^{a}	281.6^{bcd}	353.8^{ab}
**PLAPOW**	84.3^{a}	137.5^{b}	9.31^{a}	1414.6^{b}	0.53^{b}	753.2^{abc}	671.8^{b}	29.8^{a}	24.7^{a}	0.41^{a}	287.4^{abcd}	365.0^{ab}
**RUE**	84.3^{a}	137.5^{b}	6.56^{c}	1556.1^{a}	0.52^{b}	812.4^{a}	603.6^{b}	25.8^{bc}	20.3^{bc}	0.39^{a}	314.0^{a}	400.1^{a}
**FRTRL**	84.3^{a}	137.3^{b}	6.39^{c}	1292.6^{b}	0.54^{b}	705.5^{bc}	602.1^{b}	23.5^{e}	17.7^{d}	0.43^{a}	260.1^{d}	331.6^{b}
**PDHI**	84.3^{a}	137.5^{b}	6.39^{c}	1308.6^{b}	0.54^{b}	700.6^{bc}	616.0^{b}	23.5^{de}	17.7^{d}	0.43^{a}	265.4^{cd}	337.9^{b}
**PPSEN**	91.1^{a}	141.9^{ab}	8.09^{b}	1411.0^{b}	0.51^{b}	715.7^{bc}	669.2^{ab}	27.8^{ab}	22.3^{ab}	0.40^{a}	296.6^{ab}	378.9^{ab}
**bdANTPM**	84.3^{a}	144.7^{a}	6.39^{c}	1401.7^{b}	0.56^{a}	781.4^{ab}	660.6^{a}	23.7^{cde}	17.7^{d}	0.43^{a}	294.5^{abc}	377.3^{ab}
**SLNG**	84.3^{a}	137.5^{b}	6.11^{c}	1290.7^{b}	0.52^{b}	678.1^{c}	607.6^{b}	25.4^{cde}	19.7^{dc}	0.43^{a}	261.4^{d}	330.6^{b}
**SLNS**	84.3^{a}	137.5b	6.40^{c}	1302.0^{b}	0.52^{b}	681.9^{c}	610.6^{b}	23.5^{de}	17.7^{d}	0.43^{a}	263.1^{d}	335.1^{b}
**SNCG**	84.3^{a}	137.5^{b}	6.45^{c}	1310.8^{b}	0.53^{b}	689.4^{c}	617.2^{b}	25.7^{bcd}	20.0^{bcd}	0.42^{a}	265.5^{cd}	332.2^{b}
**SNCS**	84.3^{a}	137.5^{b}	6.47^{c}	1306.7^{b}	0.52^{b}	685.2^{c}	612.7^{b}	23.8^{cde}	18.0^{cd}	0.43^{a}	264.3^{d}	332.4^{b}
**MXNUP**	84.3^{a}	137.5^{b}	6.39^{c}	1300.2^{b}	0.52^{b}	680.0^{c}	608.8^{b}	23.6^{cde}	17.7^{d}	0.43^{a}	262.6^{d}	332.0^{b}
	**Gorgan**											
	**DtANT**	**DtHAR**	**MXLAI**	**WTOP**	**HI**	**WGRN**	**intPAR**	**NUP**	**NFERT**	**NLEACH**	**CTR**	**CIRGW**
	(d)	(d)	(m^2 m^{-2})	(g m^{-2})	(g g^{-1})	(g m^{-2})	(MJ m^{-2})	(g N m^{-2})	(g N m^{-2})	(g N m^{-2})	(mm)	(mm)
**STD**	141^{a}	195.7^{a}	6.4^{d}	1467.5^{de}	0.5^{b}	738.0^{d}	764.4^{c}	24.8^{d}	17.7^{e}	0.84^{a}	253.5^{cd}	188.1^{a}
**PHYL**	141^{a}	195.7^{a}	9.3^{b}	1598.5^{b}	0.51^{b}	814.9^{abcd}	847.5^{ab}	31.2^{b}	24.7^{b}	0.80^{a}	275.8^{bcd}	195.3^{a}
**PLAPOW**	141^{a}	195.7^{a}	12.6^{a}	1665.2^{ab}	0.51^{b}	855.7^{ab}	897.5^{a}	37.0^{a}	31.3^{a}	0.78^{a}	287.4^{ab}	200.5^{a}
**RUE**	141^{a}	195.7^{a}	6.6^{d}	1763.0^{a}	0.5^{b}	887.0^{a}	762.0^{c}	27.6^{c}	21.0^{cd}	0.77^{a}	304.1^{a}	232.7^{a}
**FRTRL**	141^{a}	195.6^{a}	6.4^{d}	1462.3^{e}	0.52^{b}	771.0^{bcd}	757.9^{c}	24.7^{d}	17.7^{e}	0.84^{a}	251.8^{d}	183.9^{a}
**PDHI**	141^{a}	195.9^{a}	6.4^{d}	1473.7^{de}	0.52^{b}	763.1^{cd}	771.9^{c}	24.9^{d}	17.7^{e}	0.84^{a}	255.3^{cd}	188.6^{a}
**PPSEN**	148.2^{a}	199.6^{a}	8.0^{c}	1587.9^{bc}	0.49^{b}	774.5^{bcd}	829.5^{abc}	29.6^{bc}	23.7^{bc}	0.85^{a}	280.8^{abc}	206.1^{a}
**bdANTPM**	141^{a}	203.1^{a}	6.4^{d}	1572.7^{bcd}	0.63^{a}	842.8^{abc}	813.2^{bc}	25.1^{d}	17.7^{e}	0.84^{a}	279.7^{abcd}	209.0^{a}
**SLNG**	141^{a}	195.7^{a}	6.3^{d}	1472.0^{de}	0.5^{b}	744.2^{d}	771.1^{c}	27.5^{c}	20.7^{d}	0.83^{a}	254.8^{cd}	187.6^{a}
**SLNS**	141^{a}	195.7^{a}	6.4^{d}	1469.4^{de}	0.5^{b}	739.8^{d}	766.5^{c}	24.9^{d}	17.7^{e}	0.84^{a}	253.9^{cd}	188.3^{a}
**SNCG**	141^{a}	195.7^{a}	6.5^{d}	1480.9^{cde}	0.5^{b}	748.8^{d}	776.8^{bc}	27.5^{c}	20.7^{d}	0.83^{a}	256.1^{cd}	188.5^{a}

**SNCS**	141^{a}	195.7^{a}	6.5^{d}	1472.2^{de}	0.5^{b}	740.9^{d}	767.7^{c}	24.9^{d}	17.7^{e}	0.84^{a}	254.3^{cd}	188.6^{a}
**MXNUP**	141^{a}	195.7^{a}	6.4^{d}	1468.5^{de}	0.5^{b}	738.6^{d}	765.0^{c}	24.9^{d}	17.7^{e}	0.83^{a}	253.6^{cd}	188.2^{a}
						**Quchan**						
	**DtANT** (d)	**DtHAR** (d)	**MXLAI** (m^2 m^{-2})	**WTOP** (g m^{-2})	**HI** (g g^{-1})	**WGRN** (g m^{-2})	**intPAR** (MJ m^{-2})	**NUP** (g N m^{-2})	**NFERT** (g N m^{-2})	**NLEACH** (g N m^{-2})	**CTR** (mm)	**CIRGW** (mm)
**STD**	203.5^{a}	259.3^{a}	5.7^{d}	1831.1^{e}	0.50^{b}	913.8^{d}	1036.1^{b}	28.1^{d}	24^{d}	0.44^{a}	454.7^{c}	527.1^{a}
**PHYL**	203.5^{a}	259.1^{a}	8.2^{b}	2034.3^{abc}	0.50^{b}	1020.9^{abc}	1172.2^{ab}	33.8^{b}	30.8^{b}	0.44^{a}	503.0^{abc}	549.0^{a}
**PLAPOW**	203.5^{a}	259.3^{a}	10.5^{a}	2146.0^{ab}	0.50^{b}	1080.4^{a}	1254.2^{a}	39.7^{a}	37.6^{a}	0.43^{a}	527.9^{ab}	569.2^{a}
**RUE**	203.5^{a}	258.7^{a}	5.9^{d}	2187.7^{a}	0.49^{b}	1075.0^{a}	1026.7^{b}	30.3^{cd}	27.2^{c}	0.40^{a}	542.0^{a}	602.7^{a}
**FRTRL**	203.5^{a}	259.1^{a}	5.7^{d}	1823.5^{e}	0.52^{b}	955.9^{bcd}	1030.6^{b}	28.0^{d}	24^{d}	0.44^{a}	452.1^{c}	524.7^{a}
**PDHI**	203.5^{a}	259.3^{a}	5.7^{d}	1827.0^{e}	0.50^{b}	922.5^{bcd}	1034.9^{b}	28.0^{d}	24^{d}	0.45^{a}	453.8^{c}	519.7^{a}
**PPSEN**	208.9^{a}	262.5^{a}	6.9^{c}	2002.0^{bcd}	0.50^{b}	999.1^{abcd}	1116.2^{ab}	31.5^{bc}	28.4^{bc}	0.45^{a}	508.6^{abc}	574.2^{a}
**bdANTPM**	203.5^{a}	266.7^{a}	5.7^{d}	1944.1^{cde}	0.62^{a}	1026.8^{ab}	1083.6^{ab}	28.2^{cd}	24^{d}	0.44^{a}	494.8^{abc}	568.1^{a}
**SLNG**	203.5^{a}	259.3^{a}	5.6^{d}	1835.8^{e}	0.50^{b}	923.0^{bcd}	1042.2^{b}	31.0^{bcd}	27.6^{c}	0.44^{a}	456.9^{c}	525.8^{a}
**SLNS**	203.5^{a}	259.3^{a}	5.7^{d}	1833.7^{e}	0.50^{b}	916.6^{cd}	1037.9^{b}	28.0^{d}	24^{d}	0.44^{a}	455.6^{c}	529.4^{a}
**SNCG**	203.5^{a}	259.3^{a}	5.8^{d}	1850.4^{de}	0.50^{b}	931.3^{bcd}	1049.2^{b}	30.4^{cd}	26.8^{cd}	0.44^{a}	460.5^{bc}	529.9^{a}
**SNCS**	203.5^{a}	259.3^{a}	5.8d	1838.2e	0.50b	918.7^{cd}	1039.8^{b}	28.0^{d}	24^{d}	0.44^{a}	456.6^{c}	529.5^{a}
**MXNUP**	203.5^{a}	259.3^{a}	5.8^{d}	1832.7^{e}	0.50^{b}	914.9^{cd}	1036.7^{b}	28.1^{d}	24^{d}	0.44^{a}	455.1^{c}	529.7^{a}
						**Karaj**						
	**DtANT** (d)	**DtHAR** (d)	**MXLAI** (m^2 m^{-2})	**WTOP** (g m^{-2})	**HI** (g g^{-1})	**WGRN** (g m^{-2})	**intPAR** (MJ m^{-2})	**NUP** (g N m^{-2})	**NFERT** (g N m^{-2})	**NLEACH** (g N m^{-2})	**CTR** (mm)	**CIRGW** (mm)
**STD**	180.4^{a}	230.5^{a}	6.3^{d}	1742.1^{d}	0.49^{b}	863.1^{d}	926.5^{b}	27.6^{d}	22^{d}	0.48^{a}	395.5^{c}	425.0^{cd}
**PHYL**	180.4^{a}	230.5^{a}	9.0^{b}	1898.7^{bc}	0.50^{b}	943.5^{abcd}	1034.5^{ab}	33.4^{b}	28.8^{b}	0.41^{a}	428.1^{b}	471.0^{abc}
**PLAPOW**	180.4^{a}	230.5^{a}	11.5^{a}	1968.3^{ab}	0.50^{b}	977.7^{abc}	1085.8^{a}	37.3^{a}	33.2^{a}	0.45^{a}	442.1^{b}	476.7^{ab}
**RUE**	180.4^{a}	230.3^{a}	6.6^{d}	2084.4^{a}	0.49^{b}	1019.3^{a}	915.0^{b}	29.4^{cd}	24.8^{cd}	0.42^{a}	471.9^{a}	510.0^{a}
**FRTRL**	180.4^{a}	230.3^{a}	6.3^{d}	1731.0^{d}	0.52^{b}	899.2^{bcd}	915.3^{b}	27.9^{cd}	22.8^{d}	0.47^{a}	391.5^{c}	424.7^{cd}
**PDHI**	180.4^{a}	230.7^{a}	6.3^{d}	1755.7^{cd}	0.51^{b}	890.6^{bcd}	940.9^{b}	27.9^{cd}	22.8^{d}	0.47^{a}	401.1^{c}	437.2^{bcd}
**PPSEN**	184.9^{a}	233.3^{a}	7.6^{c}	1844.9^{bcd}	0.48^{b}	884.1^{cd}	977.1^{ab}	30.8^{bc}	26^{bc}	0.44^{a}	430.6^{b}	476.5^{ab}
**bdANTPM**	180.4^{a}	237.7^{a}	6.3^{d}	1862.9^{bcd}	0.62^{a}	983.7^{ab}	987.8^{ab}	28.1^{cd}	22.8^{d}	0.47^{a}	440.5^{b}	488.9^{a}
**SLNG**	180.4^{a}	230.5^{a}	6.2^{d}	1735.6^{d}	0.49^{b}	860.3^{d}	925.5^{b}	29.3^{cd}	24^{cd}	0.47^{a}	394.1^{c}	419.0^{d}
**SLNS**	180.4^{a}	230.5^{a}	6.3^{d}	1743.5^{d}	0.49^{b}	864.5^{d}	928.0^{b}	27.6^{d}	22^{d}	0.48^{a}	396.0^{c}	425.1^{cd}
**SNCG**	180.4^{a}	230.5^{a}	6.3^{d}	1748.1^{d}	0.50^{b}	867.8^{d}	932.9^{b}	29.6c^{d}	24.4^{cd}	0.47^{a}	397.2^{c}	423.8^{cd}

**SNCS**	180.4^{a}	230.5^{a}	6.4^{d}	1746.1^{d}	0.50^{b}	865.9^{d}	929.5^{b}	27.8^{cd}	22.4^{d}	0.47^{a}	396.6^{c}	423.4^{cd}
**MXNUP**	180.4^{a}	230.5^{a}	6.3^{d}	1743.6^{d}	0.50^{b}	864.6^{d}	928.2^{b}	28.2^{cd}	22.8^{d}	0.47^{a}	396.0^{c}	423.0^{cd}

Table S4. Correlation coefficients among various pairs of crop characteristics: days to anthesis (DtANT), days to harvest maturity (DtHAR), maximum LAI (MXLAI), total dry matter (WTOP, g m^{-2}), harvest index (HI, g g^{-1}), grain dry matter (WGRN, g m^{-2}), intercepted PAR (intPAR, Mj m^{-2}), nitrogen accumulation in above-ground organs (NUP, g N m^{-2}), applied N fertilizer (NFERT, g N m^{-2}), cumulative nitrogen leached from the soil (NLEACH, g N m^{-2}), Cumulative transpiration (CTR, mm) and Cumulative applied irrigation water (CIRGW, mm).

	**DtANT**	**DtHAR**	**MXLAI**	**WTOP**	**HI**	**WGRN**	**intPAR**	**NUP**	**NFERT**	**NLEACH**	**CTR**	**CIRGW**
**DtANT**	1.00											
**DtHAR**	1.00***	1.00										
**MXLAI**	-0.10	-0.11	1.00									
**WTOP**	0.90***	0.90***	0.11	1.00								
**HI**	-0.62***	-0.60***	-0.07	-0.53***	1.00							
**WGRN**	0.87***	0.87***	0.10	0.98***	-0.39**	1.00						
**intPAR**	0.96***	0.96***	0.11	0.93***	-0.53***	0.92***	1.00					
**NUP**	0.57***	0.55***	0.70***	0.72***	-0.42**	0.70***	0.73***	1.00				
**NFERT**	0.62***	0.61***	0.59***	0.79***	-0.37**	0.77***	0.79***	0.98***	1.00			
**NLEACH**	-0.08	-0.07	0.08	-0.26	-0.32*	-0.30*	-0.19	-0.15	-0.32*	1.00		
**CTR**	0.85***	0.85***	-0.04***	0.93***	-0.34*	0.93***	0.90***	0.61***	0.74***	-0.54***	1.00	
**CIRGW**	0.61***	0.61***	-0.12	0.73***	-0.06	0.76***	0.69***	0.43**	0.61***	-0.82***	0.92***	1.00

*, ** and *** are significant at the 0.05, 0.01 and 0.001 level of probability, respectively
